# Elucidation of the preferred routes of C8-vinyl reduction in chlorophyll and bacteriochlorophyll biosynthesis

**DOI:** 10.1042/BJ20140163

**Published:** 2014-08-22

**Authors:** Daniel P. Canniffe, Jack W. Chidgey, C. Neil Hunter

**Affiliations:** *Department of Molecular Biology and Biotechnology, University of Sheffield, Firth Court, Western Bank, Sheffield S10 2TN, U.K.

**Keywords:** bacteriochlorophyll biosynthesis, chlorophyll, C8-vinyl reductase, photosynthesis, *Rhodobacter sphaeroides*, *Synechocystis* sp. PCC6803, BChl, bacteriochlorophyll, Chl, chlorophyll, Chlide, chlorophyllide, COR, Chlide *a* oxidoreductase, DPOR, dark-operative POR, 8E, C8-ethyl, Pchlide, protochlorophyllide, POR, Pchlide oxidoreductase, 8V, C8-vinyl, 8VR, 8V reductase, WT, wild-type

## Abstract

Most of the chlorophylls and bacteriochlorophylls utilized for light harvesting by phototrophic organisms carry an ethyl group at the C8 position of the molecule, the product of a C8-vinyl reductase acting on a chlorophyll/bacteriochlorophyll biosynthetic precursor. Two unrelated classes of C8-vinyl reductase are known to exist, BciA and BciB, found in the purple phototroph *Rhodobacter sphaeroides* and the cyanobacterium *Synechocystis* sp. PCC6803 respectively. We constructed strains of each bacterium with the native C8-vinyl reductase swapped for the other class of the enzyme, and combined these replacements with a series of deletions of the native *bch* and *chl* genes. *In vivo* data indicate that the preferred substrates for both classes of the enzyme is C8-vinyl chlorophyllide, with C8-vinyl protochlorophyllide reduced only under conditions in which this pigment accumulates as a result of perturbed formation of chlorophyllide.

## Abstract 2

The step in (bacterio)chlorophyll biosynthesis at which the vinyl group at the C8 position is reduced, forming an ethyl group, has been disputed. Results from species utilizing unrelated reductases suggest that C8-vinyl chlorophyllide is the preferred substrate for both enzymes.

## INTRODUCTION

Photosynthesis, the process by which solar energy is converted into chemical potential energy, is dependent on light-capturing BChl (bacteriochlorophyll)/Chl (chlorophyll) pigments, incorporated within antenna complexes of plants, algae and phototrophic bacteria. The spectral range of these complexes is extended by modifications to the (B)Chl macrocycle, which influence pigment–pigment and pigment–protein interactions within the antenna complexes [1–3]. Among these modifications is the pre-sence of ethyl and vinyl groups, which can extend or confine the delocalized π-electron system of the (B)Chl macrocycle [2].

**Figure 1:**
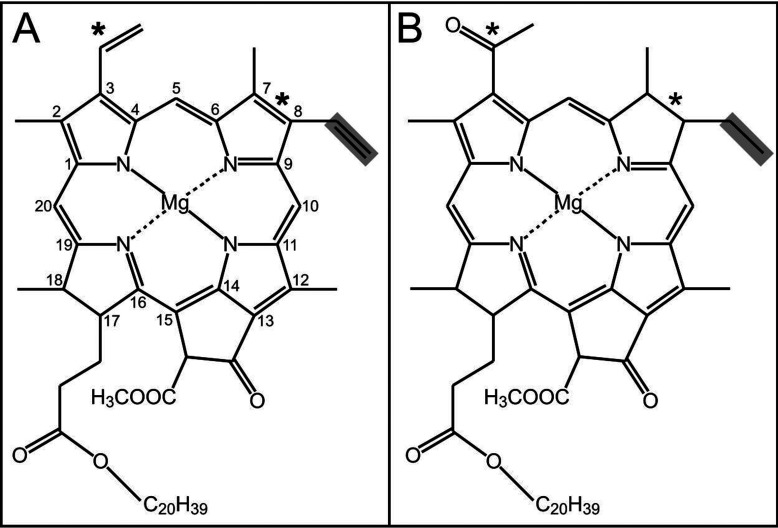
Chemical structures of (B)Chls *a* (**A**) IUPAC numbered chemical structure of Chl *a* with a vinyl group at the C8 position (highlighted) and (**B**) chemical structure of BChl *a* with an ethyl group at the C8 position (highlighted), having been reduced by an 8V reductase. The structural differences between Chl *a* and BChl *a* are indicated by asterisks.

The majority of (B)Chls utilized for light-harvesting carry an ethyl group at the C8 position (8E) of the macrocycle (Figure 1). This group is produced by the reduction of a vinyl group (8V), catalysed by an 8VR (8V reductase; EC 1.3.1.75), resulting in the production of an 8E pigment. The original linear pathway for the biosynthesis of Chl was proposed by Granick [4]; this sequence of reactions placed the reduction of the 8V group after the formation of the isocyclic E ring of Pchlide (protochlorophyllide), catalysed by a cyclase, and before the reduction of the C17=C18 double bond by a POR (Pchlide oxidoreductase), yielding Chlide (chlorophyllide), which is converted into the mature pigment by Chl synthase. However, many 8E and 8V Chl precursors have been detected in plants and algae that cannot be accounted for by the stepwise operation of a linear biosynthetic pathway [5–7]. The ratios of these 8E and 8V intermediates can vary depending on the species, tissue type and growth conditions [6,8–10].

The analysis of mutants in the AT5G18660 gene of *Arabidopsis thaliana* demonstrated the accumulation of 8V rather than 8E Chls [11,12]. The recombinant protein encoded by this gene, produced in *Escherichia coli*, was able to reduce 8V Chlide to its 8E form, confirming that the gene product was a functional 8VR. Subsequently, orthologues of this gene in rice [13], the green sulfur bacterium *Chlorobaculum tepidum* [14] and the purple non-sulfur bacterium *Rhodobacter sphaeroides* [15] were shown to encode 8VR enzymes.

The genomes of many freshwater cyanobacteria do not contain orthologues of this 8VR although they utilize 8E Chl, indicating the existence of a second 8VR (termed BciB) unrelated to the BciA first identified in *A. thaliana*. Two studies of the cyanobacterium *Synechocystis* sp. PCC6803 (*Synechocystis*) showed that mutants in the slr1923 ORF were unable to grow under high-light conditions and accumulated 8V rather than 8E Chl [16,17]. Subsequently, an slr1923 orthologue (Ctha_1208) from the green sulfur bacterium *Chloroherpeton thalassium* was shown to complement the *C. tepidum* Δ*bciA* mutant, recovering the synthesis of 8E (B)Chls, demonstrating the activity of the second, BciB, class of 8VRs [18].

In a reciprocal experiment, BciA from *R. sphaeroides* was able to complement the Δ*bciB* (Δslr1923) mutant of *Synechocystis*, although the Δ*bciA* mutant of *R. sphaeroides* was still able to synthesize 8E BChl [15], suggesting the existence of a third class of 8VR. Tsukatani et al. [19] have now demonstrated that the COR (Chlide *a* oxidoreductase) from the related BChl *a*-utilizing bacterium *Rhodobacter capsulatus*, which reduces the C7=C8 double bond of Chlide, is also able to reduce the 8V group of Chlide [19]. Thus the third class of 8VR has now been discovered, referred to in the present paper as a COR-type reductase. *R. capsulatus* also contains an orthologue of *bciA* (the translated sequence of which is 61% identical and 72% similar with BciA from *R. sphaeroides*). It is likely that organisms with 8E-BChls use COR to reduce the 8V group of any Chlide molecules that have bypassed the conventional 8VR. This mechanism may also account for the lack of any *bciA* or *bciB* orthologues in the genomes of 8E BChl *a*-producing *Roseiflexus* species [20] of green non-sulfur bacteria.

*In vitro* assays performed with BciA-type enzymes from various species have demonstrated substrate flexibility within this class, as well as the requirement of NADPH as a reductant [13,14,21,22]. The first study of *in vitro* activity of a BciB-type 8VR showed that the enzyme from *Chp. thalassium* can reduce the 8V group of Pchlide in the presence of FAD, and that the active protein contains two [4Fe–4S] clusters [23].

In the present study, we analyse various *R. sphaeroides* and *Synechocystis* strains that lack their native conventional 8VRs, and heterologously express *bciA* or *bciB* genes from the reciprocal organism. Our results indicate that the preferred substrate for both BciA and BciB is 8V Chlide, and that 8V Pchlide is reduced only under conditions in which this pigment accumulates predominantly over Chlide.

## MATERIALS AND METHODS

### Growth of described strains

*R. sphaeroides* strains were grown semi-aerobically in the dark in a rotary shaker at 34°C in liquid M22+ medium [24] supplemented with 0.1% casamino acids.

*Synechocystis* strains were grown photomixotrophically (with light, CO_2_ and a pre-fixed carbon source) in a rotary shaker under low (5 μmol of photons·m^−2^·s^−1^), moderate (50 μmol of photons·m^−2^·s^−1^) or high (250 μmol of photons·m^−2^·s^−1^) light conditions at 30°C in liquid BG-11 medium [25] supplemented with 5 mM glucose and 10 mM TES (pH 8.2).

*E*. *coli* strains JM109 [26] and S17-1 [27] transformed with pK18*mobsacB* plasmids were grown in a rotary shaker at 37°C in LB medium supplemented with 30 μg/ml kanamycin. All strains and plasmids used in the present study are listed in Supplementary Table S1 (http://www.biochemj.org/bj/462/bj4620433add.htm).

### Construction of mutants of *R. sphaeroides*

*R. sphaeroides* genes were deleted using the allelic exchange vector pK18*mobsacB* [28]. Sequences up- and down-stream of target genes were amplified with the relevant UpF and UpR and DownF and DownR primers respectively (the overlapping genes *bchC* and *bchX* were deleted together). Sequences of all of the primers used in the present study can be found in Supplementary Table S2 (http://www.biochemj.org/bj/462/bj4620433add.htm). The up- and down-stream PCR products were digested with the relevant restriction enzymes and ligated into cut pK18*mobsacB*. Sequenced clones were conjugated into *R. sphaeroides* from *E. coli* S17-1, and transconjugants in which the clone had integrated into the genome by homologous recombination were selected on M22+ medium supplemented with kanamycin. Transconjugants that had undergone a second recombination event were then selected on M22+ supplemented with 10% (w/v) sucrose, lacking kanamycin. Sucrose-resistant kanamycin-sensitive colonies had excised the allelic exchange vector through the second recombination event [29]. The deletion of the desired gene was confirmed by colony PCR using relevant CheckF and CheckR primers.

Strains harbouring the *Synechocystis bciB* gene (slr1923) were created as follows; slr1923 was amplified from WT (wild-type) *Synechocystis* genomic DNA using 1923INDF and 1923INDR primers, digested and cloned into the BamHI/HindIII sites of pIND4 [30]. The resulting sequenced plasmid was conjugated into relevant strains from *E. coli* S17-1 and transconjugants were selected on M22+ medium supplemented with kanamycin.

### Expression of *bciB* in *R. sphaeroides*

Expression of *Synechocystis bciB* (slr1923) from pIND4[*bciB*] was induced in *R. sphaeroides* cultures at *D*_680_ 0.8 by the addition of IPTG at a final concentration of 100 μM. Samples from these cultures were taken 3 h after induction.

### Construction of deletion mutant of *Synechocystis*

Replacement of the *Synechocystis chlB* gene (slr0772) with a zeocin resistance cassette was achieved using a modified megaprimer mutagenesis method [31]. Sequences of approximately 400 bp up- and down-stream of *chlB* were amplified using the chlB UpF and UpR and DownF and DownR primers respectively, to generate primary megaprimers. The upstream reverse and downstream forward primers contained overhang sequences able to amplify the zeocin resistance cassette from the pZEO plasmid. The upstream megaprimer, along with the zeo^R^R primer, and the downstream megaprimer with the zeo^R^F primer were used to amplify large overlapping portions of the resistance cassette. These resulting secondary megaprimers were then used for overlap extension PCR to generate the final mutagenesis fragment. This fragment was transformed into WT *Synechocystis* and transformants were selected on BG-11 agar plates containing 2 μg/ml zeocin and fully segregated by incrementally doubling the concentration of antibiotic to 16 μg/ml. Construction of a fully segregated strain was confirmed by colony PCR using the UpF and DownR primers used to generate the primary megaprimers.

### Extraction of pigments

Pigments were extracted from cell pellets after washing in 20 mM Hepes (pH 7.2) by adding nine pellet volumes of 0.2% ammonia in methanol, vortex-mixing for 30 s and incubating on ice for 20 min. The extracts were clarified by centrifugation (15000 ***g*** for 5 min at 4°C) and the supernatants were immediately analysed on an Agilent 1200 HPLC system. Chl *a* and carotenoids were removed from clarified extracts of *Synechocystis* strains by the addition of one pellet volume of 5 M NaCl and four pellet volumes of hexane. The samples were mixed and allowed to partition, the upper hexane phase was discarded and the lower methanol phase was clarified before analysis.

### Analysis of pigments by HPLC

Pchlide and Chlide species were separated on a YMC30 C_30_ reverse-phase column (3 μm particle size; 250 mm×4.6 mm) using a method modified from that of Kruk and Myśliwa-Kurdziel [32]. Solvents A and B were 10:90 (v/v) methanol/500 mM ammonium acetate or methanol respectively. Pigments were eluted at 1 ml/min at 40°C on a linear gradient of 82–98% solvent B over 40 min, increasing to 100% to wash the column. Elution of Pchlide and Chlide species was monitored by checking absorbance at 632 nm and 665 nm respectively.

Chl *a* species were separated on a Phenomenex Aqua C_18_ reverse-phase column (5 μm particle size; 125 Å pore size; 250 mm×4.6 mm) using a method modified from that of van Heukelem et al. [33]. Solvents A and B were 80:20 (v/v) methanol/500 mM ammonium acetate and 80:20 (v/v) methanol/acetone respectively. Pigments were eluted at 1 ml/min at 40°C on a linear gradient of 92–94% solvent B over 25 min, increasing to 100% to wash the column. Elution of Chl *a* species was monitored by checking absorbance at 665 nm.

## RESULTS

### BciA preferentially reduces Chlide in *R. sphaeroides*

**Figure 2:**
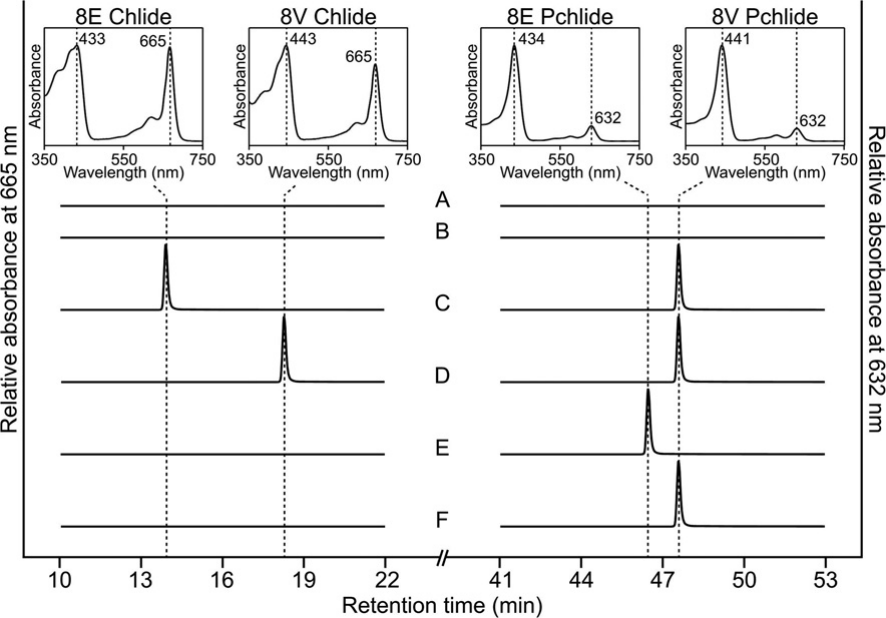
HPLC elution profiles of pigments extracted from *R. sphaeroides* strains HPLC elution profiles of extracts from pellets of (A) WT, (B) Δ*bciA*, (C) Δ*bchCXF*, (D) Δ*bchCXF*/Δ*bciA*, (E) V3 and (F) V3/Δ*bciA* strains. Retention times and Soret maxima of peaks are used to identify pigment species (inset). Traces are normalized to major peak height for clarity.

Our previous study on BciA in *R. sphaeroides* indicated that a second unrelated 8VR was active in reducing a BChl precursor, compensating for the loss of BciA [15]. Subsequently, Tsukatani et al. [19] discovered that COR, which catalyses the reduction of the C7=C8 double bond, was also able to reduce the 8V group. This dual function of COR, encoded by the *bchX*, *bchY* and *bchZ* genes, accounts for the lack of a mutant phenotype in our previously described Δ*bciA* strain. Therefore, in order to determine the preferred substrate of the conventional 8VR in *R. sphaeroides*, mutants in several genes essential for (B)Chl biosynthesis were constructed in both WT and Δ*bciA* backgrounds. The pigments accumulated in these strains were extracted from pellets of semi-aerobically grown cultures and analysed by HPLC (Figure 2). Neither the WT (Figure 2, A) nor Δ*bciA* (Figure 2, B) accumulates BChl precursors in measurable amounts. A mutant in which the steps exclusive to BChl biosynthesis are blocked, Δ*bchCXF*, accumulated 8E Chlide and 8V Pchlide (Figure 2, C), whereas the 8V forms of both Chlide and Pchlide accumulated following additional deletion of *bciA* (Figure 2, D). These data indicate that before any 8V reduction by COR, BciA will reduce only 8V Chlide, as 8E Pchlide is not detected. Interestingly, the V3 mutant of *R. sphaeroides*, an unmapped mutant in a subunit of DPOR (dark-operative POR) [34], accumulates Pchlide reduced at the C8 position (Figure 2E), whereas this strain lacking *bciA* accumulates 8V Pchlide (Figure 2F). These data indicate that BciA will reduce the 8V group of Pchlide, but only in the absence of Chlide.

In order to test whether the unrelated 8VR from *Synechocystis*, BciB, was able to complement the loss of *bciA*, the encoding gene (slr1923) was expressed from pIND4[*bciB*] in each of the described *R. sphaeroides* strains. Although presence of the re-combinant protein could be detected by immunoblotting (Supplementary Figure S1 at http://www.biochemj.org/bj/462/bj4620433add.htm), no recovery of 8V reduction was observed in any strain (results not shown).

### BciB, as well as recombinant BciA, preferentially reduces Chlide in *Synechocystis*

**Figure 3:**
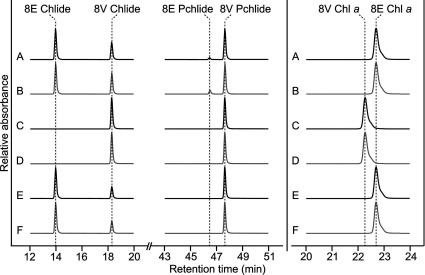
Typical HPLC elution profiles of pigments extracted from *Synechocystis* strains HPLC elution profiles of extracts from pellets of WT (A and B), Δ*bciB* (C and D) and Δ*bciB*::*bciA^Rs^* (E and F) strains. Strains were grown under moderate light (black) or low light (grey). Separated precursor Pchlide and Chlide species are displayed on the left-hand side of the Figure and separated Chl *a* species are displayed on the right-hand side. Traces are normalized to major peak height for clarity.

In order to determine the preferred substrate of the native 8VR in *Synechocystis*, WT, Δ*bciB* and Δ*bciB*::*bciA^Rs^* strains were examined. In this last case, *R. sphaeroides bciA* was integrated into the *Synechocystis* genome, which compensates for the loss of *bciB* [15]. Strains were grown photomixotrophically at moderate and low light intensities, which results in the accumulation of low levels of intracellular precursor pigments; high light or photoautotrophic growth under all tested light intensities resulted in undetectably low levels of precursor pigments. The composition of each was analysed by HPLC (Figure 3). When the WT was grown photomixotrophically under moderate light intensity 96% of the Pchlide species detected were the non-reduced 8V form, with the 8E pigment accounting for only 4%, whereas the majority (63%) of the accumulated Chlide was found to be reduced (Figure 3, A). Levels of reduced Pchlide increased to 9% under low light (Figure 3B), whereas the Chlide pool was composed of 59% reduced and 41% non-reduced pigment respectively. Under both light regimes, only fully reduced Chl *a* was detected. As expected, *bciB* deletion abolished formation of 8E pigments, and the 8V forms of Pchlide, Chlide and Chl *a* accumulated under photomixotrophic conditions (Figure 3, C and D). The *Synechocystis* Δ*bciB*::*bciA^Rs^* strain, which produces 8E Chl *a* [15], was also analysed using photomixotrophically grown cells; non-reduced 8V Pchlide accumulated at both moderate (Figure 3, E) and low (Figure 3, F) light, whereas 70% and 67% respectively of Chlide species detected were the reduced 8E form of the pigment.

The *chlB* gene encoding a subunit of the ‘dark’ DPOR enzyme was deleted in the WT, Δ*bciB* and Δ*bciB*::*bciA^Rs^* backgrounds, leaving the light-activated form of POR as the only enzyme able to reduce the C17=C18 double bond. These strains were grown photomixotrophically at low light until reaching a *D*_740_ of 0.5 before illumination was halted for 16 h. Under these conditions the strains were unable to reduce any Pchlide present in the cells to Chlide, halting Chl biosynthesis and mimicking the con-ditions of etiolated plant tissue. The majority, 61%, of the Pchlide extracted from the Δ*chlB* strain was in the 8E form, with 39% being the non-reduced pigment (Supplementary Figure S2, A at http://www.biochemj.org/bj/462/bj4620433add.htm). As expected, the Δ*chlB*/Δ*bciB* strain accumulated 8V Pchlide only (Supplementary Figure S2, B); partial complementation of the Δ*bciB* mutation was observed in the Δ*chlB*/Δ*bciB*::*bciA^Rs^* strain, with 42% 8E Pchlide and 58% in the 8V form (Supplementary Figure 2, C). The pigments of these strains lacking *chlB* did not differ significantly from those containing the functional DPOR when grown under illumination (results not shown).

### Localization of native and recombinant 8VRs in *Synechocystis*

**Figure 4:**
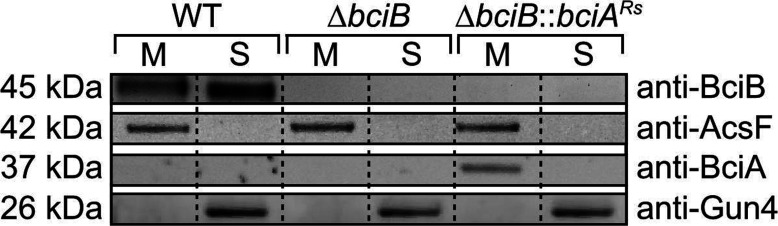
Detection of 8VRs in *Synechocystis* by Western blotting Separated membrane (M) and soluble (S) fractions from described strains of *Synechocystis*, resolved by SDS/PAGE and transferred on to a membrane were probed with anti-BciA and anti-BciB antibodies. The membrane was also probed with antibodies to known membrane-associated and soluble Chl biosynthetic proteins AcsF and Gun4 respectively.

To determine the localization of BciB in *Synechocystis*, as well as the recombinant BciA, samples from cultures of WT, Δ*bciB* and Δ*bciB*::*bciA^Rs^* grown under moderate light intensity were disrupted by bead-beating and the soluble and membrane fractions were separated by centrifugation. These samples were resolved by SDS/PAGE and transferred on to a PVDF membrane which was probed with antibodies raised against BciB (*Synechocystis*) and BciA (*A. thaliana*), as well as those raised against known membrane-associated (AcsF, *A. thaliana* CHL27; Agrisera) and soluble (Gun4, *Synechocystis*) proteins involved in Chl biosynthesis (Figure 4). The blot indicates that the native BciB protein is found in the cytoplasm as well as being localized to the thylakoid membrane, whereas the recombinant BciA is only detected in the membrane fraction.

## DISCUSSION

The linear biosynthetic pathway for the biosynthesis of Chl, first proposed by Granick [4], suggested that 8V Pchlide was the substrate for the 8VR, which catalysed the reduction of the 8V group producing 8E Pchlide. Studies subsequently performed on etiolated plant tissue, in which various Chl precursor molecules carrying reduced C8 groups were detected, indicated that the 8VR acted upon multiple substrates, refuting the proposed linear nature of the pathway [5–7]. Nevertheless, linear pathways are frequently used to describe the steps of (B)Chl biosynthesis [35,36]. Contemporary *in vitro* studies on recombinant BciAs have reinforced the notion of substrate flexibility within this class of enzyme. Nagata et al. [21] demonstrated that Chlide *a* is the preferred substrate of the *A*. *thaliana* enzyme, with no reduction of 8V groups of magnesium protoporphyrin IX, Pchlide, Chlide *b*, Chl *a* and Chl *b* detected. The related enzyme from rice, while not tested with magnesium protoporphyrin IX or Pchlide, was able to reduce the 8V forms of both Chlide *a* and Chl *a* to their 8E forms [13]. BciA from the green sulfur bacterium *C. tepidum* was shown to catalyse the reduction of 8V Pchlide [14]. More recently, a substantial *in vitro* study of the reductive activities of BciAs from maize and cucumber, as well as those from rice and *A. thaliana*, on the 8V forms of Chl *a*, Chlide *a*, Pchlide, magnesium protoporphyrin monomethyl ester and magnesium protoporphyrin IX, revealed that the rice and maize enzymes could reduce all of the tested substrates. The cucumber and *A. thaliana* proteins were able to efficiently reduce 8V Chlide, with 8E forms of Pchlide and magnesium protoporphyrin monomethyl ester being detected after 10 h incubations under assay conditions [22]. In the case of each enzyme, the assays containing 8V Chlide demonstrated the fastest reaction rates.

The present study on *R. sphaeroides* suggests that 8V Chlide is the preferred substrate for BciA in this organism. When the steps exclusive to BChl biosynthesis are removed (Δ*bchCXF*), preventing the 8V reduction catalysed by the COR-type reductase, both Pchlide and Chlide accumulate in the mutant. However, only Chlide is present in its reduced 8E form, indicating that *R. sphaeroides* BciA cannot reduce 8V Pchlide. Interestingly, in a mutant blocked at Pchlide, V3, the accumulated pigment carries a reduced C8 group, whereas this pigment remains in the 8V form when *bciA* is deleted from this mutant. These data indicate that BciA can reduce both Pchlide and Chlide, but will not reduce Pchlide when Chlide is present, potentially due to the enzyme having a lower *K*_m_ value for Chlide than Pchlide. This observation indicates that, although BciA demonstrates flexible specificity, biosynthetic heterogeneity does not occur in this organism when its preferred substrate is present, implying that, in this case, the biosynthetic pathway is linear.

The expression of *Synechocystis bciB* in the described strains of *R. sphaeroides* lacking *bciA* was unable to restore the accumulation of pigments containing groups reduced at the C8 position. The previously described complementation of a *bciA* mutant of *C. tepidum* with *bciB* from the green sulfur bacterium *Chp. thalassium*, resulting in the strain regaining the ability to reduce 8V groups [18], as well as the recent publication of an *in vitro* assay using recombinant BciA from this organism [23], demonstrate that BciB functions as an 8VR and does not require the presence of an additional subunit. The recombinant protein is found in the membrane fraction of cells expressing *bciB*. Proteomic analysis of *R. sphaeroides* has indicated that enzymes involved in BChl biosynthesis are membrane-localized [37], therefore the lack of activity of the recombinant enzyme in the present study may be due to misfolding of the protein upon expression, the inability to interact with native BChl biosynthesis enzymes, or the lack of available reductant in this host.

Unlike purple bacteria such as *R. sphaeroides* that can grow chemotrophically under semi-aerobic conditions in the dark, allowing deletion of genes essential for (B)Chl biosynthesis, *Synechocystis* is an obligate phototroph, and thus maintenance of Chl biosynthesis is imperative for viability. Under photoautotrophic growth conditions, Chl precursors do not accumulate in greater than trace amounts in this organism, possibly due to their phototoxic nature. Therefore, in order to induce accumulation of Chl precursors, the strains described in the present study were grown photomixotrophically. Analysis of the pigments accumulated in the WT strain indicate that the majority of Pchlide exists in the non-reduced form; species carrying an 8E group range between only 4% of the total Pchlide at moderate light and 9% at low light, whereas the majority of extracted Chlide appears to be reduced, ranging between 59 and 63% in low and moderate light. These data indicate that, although the native BciB is able to reduce the 8V group of Pchlide, the preferred substrate for this enzyme is 8V Chlide, with only a small amount of 8E Pchlide detected in the WT when grown under pigment accumulating conditions.

In the complemented Δ*bciB*::*bciA^Rs^* strain, which we have previously demonstrated to be able to produce 8E Chl under photoautotrophic conditions [15], only the non-reduced form of Pchlide could be detected in cells grown photomixotrophically. However, as with the WT, the 8E form of Chlide was the predominant species under both moderate and low light. These data lend further support for 8V Chlide being the preferred substrate for the *R. sphaeroides* enzyme.

A previously described mutant of *Synechocystis* in which the gene encoding light-activated POR and *chlL*, encoding a subunit of DPOR, were deleted in a photosystem I-less background was able to produce small amounts of mature Chl under weak continuous illumination [38]. The authors proposed that this completion of the biosynthetic pathway occurred via the remaining catalytic ChlB/ChlN subunits of DPOR. In creating a more faithful cyanobacterial model of etiolated plant tissue, we deleted *chlB* in WT, Δ*bciB* and Δ*bciB*::*bciA^Rs^* backgrounds, leaving the light-activated enzyme as the only functional POR in the strains. Analysis of the pigments accumulated in these strains, grown under low light and transferred to the dark, demonstrated that both native BciB and recombinant BciA are able to reduce accumulated Pchlide when Chl biosynthesis is halted at this step. The native enzyme was more efficient at reducing the accumulated pigment than BciA, resulting in 61% of total detected Pchlide being the reduced form, compared with 42% in the complemented strain. In comparison, the previously described *chlL*/*por* mutant in the photosystem I-less background accumulated intracellular and excreted extracellular pools of Pchlide consisting of 80% 8E and 20% 8V forms [38].

These data suggest that, as with BciA in *R. sphaeroides*, BciB is able to reduce 8V forms of both Pchlide and Chlide, with Chlide being preferentially reduced and Pchlide being reduced only when it accumulates as a result of increased flux down the Chl biosynthetic pathway in photomixotrophically grown cells. Unlike in *R. sphaeroides*, the native BciB 8VR is shown to reduce some Pchlide in the presence of Chlide, which suggests that a slightly lower specificity for its preferred substrate. Recombinant BciA*^Rs^* does not appear to reduce Pchlide when Chlide is being formed, possibly because of its higher specificity for Chlide, compared with BciB. Interestingly, BciB is found localized to both the thylakoid membrane and soluble fraction, whereas recombinant BciA*^Rs^* is only detected in the membrane fraction. These findings may explain why no reduction in Pchlide by BciA*^Rs^* was observed when the pathway was functional; it is possible that the small amount of reduced Pchlide detected in WT cultures was formed by the native BciB enzyme found in the soluble fraction. BciB could have a lower specificity than BciA*^Rs^* for Chlide and a greater tendency to use Pchlide as a substrate.

**Figure 5:**
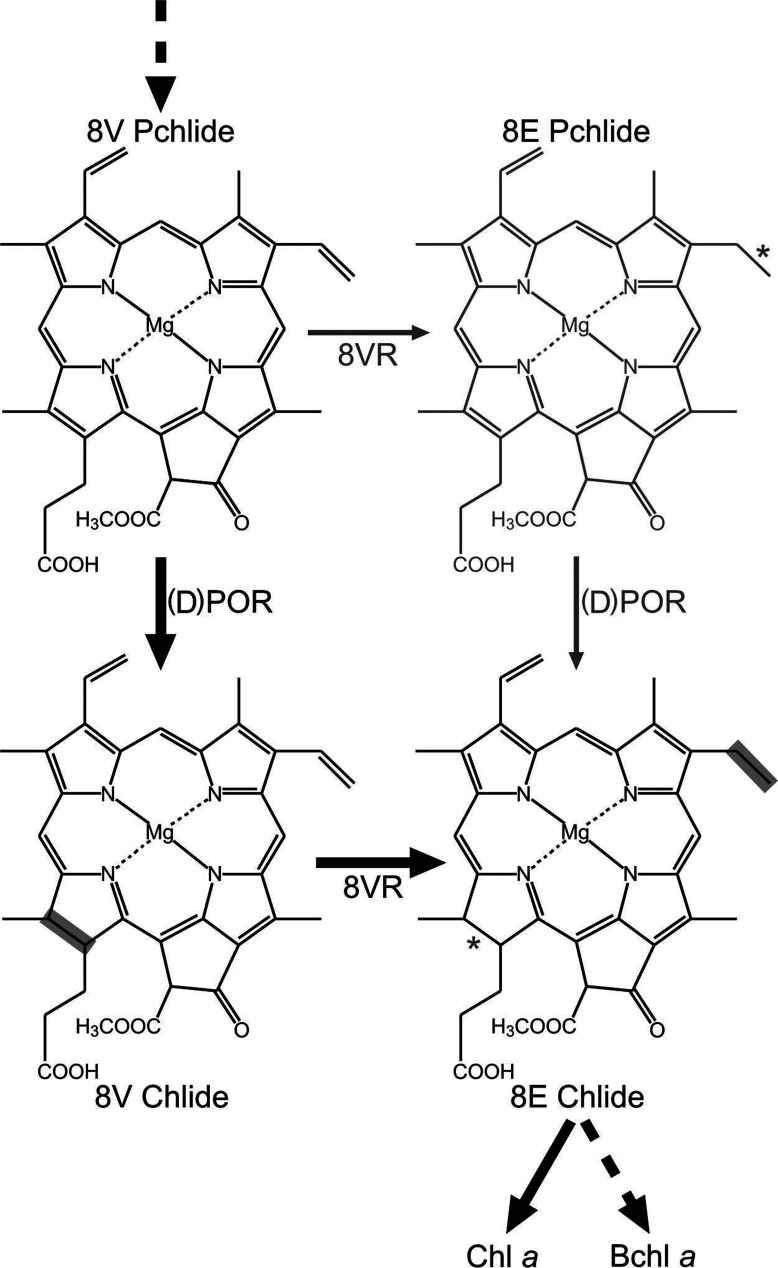
Routes of conventional 8V reduction observed *in vivo* Black arrows delineate the preferred route of (B)Chl biosynthesis, with reduced bonds highlighted by boxes. Grey arrows illustrate the route taken under conditions in which 8V Pchlide is accumulated, with reduced bonds highlighted by asterisks. Dashed arrows indicate more than one enzymatic step.

In order to avoid the accumulation of unbound phototoxic pigments, metabolic channelling of precursor molecules, facilitated by interaction between neighbouring enzymes, has been proposed [39,40]. The data presented here lead us to concur that (B)Chl biosynthesis appears to be a stepwise pathway in both *R. sphaeroides* and *Synechocystis*, under conditions where mature pigment formation is unperturbed (Figure 5). The reduction in Pchlide in both species when (B)Chl biosynthesis is stopped at this pigment is also consistent with results previously obtained from etiolated plant tissue [5–10].

Many strains of green sulfur bacteria appear to employ multiple conventional 8VRs for (B)Chl biosynthesis, either containing genes encoding enzymes of both classes (e.g. *Prosthecochloris aestuarii*) or more than one copy of *bciB* (e.g. *Chlorobium phaeobacteroides*) [18]; these different 8VRs possibly act at different steps of the pathway when present in the same cell. However, the activities of multiple conventional 8VRs from the same organism have yet to be demonstrated, although the combination of a conventional and COR 8VRs in the same pathway does operate in *R. sphaeroides*. The genomes of many plant species including *A. thaliana* and rice, which rely on BciA for 8V group reduction, contain orthologues of *bciB* which appeared to have become redundant in these species. However, Meguro et al. [41] demonstrated that the *bciB* orthologue in *A. thaliana* encodes an enzyme involved in the conversion of Chl *b* back into Chl *a*, a process important for greening, acclimation to light intensity and senescence in higher plants. This enzyme is proposed to have evolved from a diatom BciB, and now catalyses a new step in pigment biosynthesis [41]. It will be of interest to discover whether the photosynthetic bacteria containing more than one 8VR orthologue demonstrate redundancy in these genes or employ multiple 8VRs for the production of fully reduced mature (B)Chls.

## Online data

Supplementary data
