# Supplementary material for: Elucidation of the preferred routes of C8-vinyl reduction in chlorophyll and bacteriochlorophyll biosynthesis
Source: Biochem J. 2014 Aug 22;462(Pt 3):433–40. doi: 10.1042/BJ20140163 (PMC4214422; doi:10.1042/BJ20140163)
Supplement: Supplementary data [file bj4620433add.pdf]

Biochem. J. (2014) **462**, 433–440 (Printed in Great Britain) doi:10.1042/BJ20140163

SUPPLEMENTARY ONLINE DATA

Elucidation of the preferred routes of C8-vinyl reduction in chlorophyll and bacteriochlorophyll biosynthesis

Daniel P. CANNIFFE*[1], Jack W. CHIDGEY* and C. Neil HUNTER*

*Department of Molecular Biology and Biotechnology, University of Sheffield, Firth Court, Western Bank, Sheffield S10 2TN, U.K.

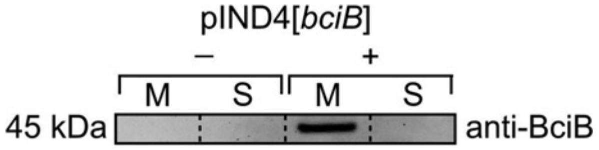

Figure S1 Expression of recombinant *bciB* in *R. sphaeroides* detected by Western blotting

Separated membrane (M) and soluble (S) fractions of Δ*bchCXF*/Δ*bciA* lacking (−) and containing (+) pIND4[*bciB*] grown under expression-inducing conditions were separated by SDS/PAGE and transferred to a membrane that was probed with an anti-BciB antibody.

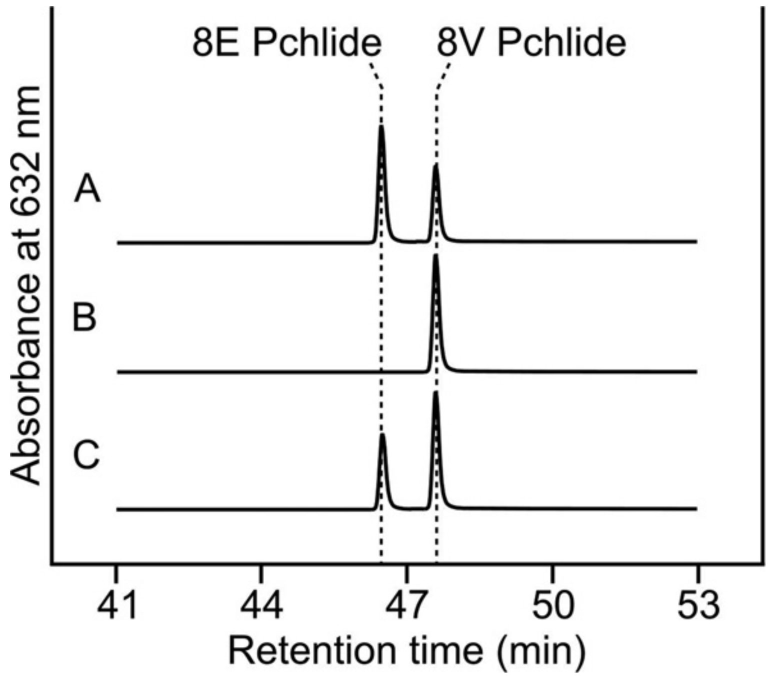

Figure S2 HPLC elution profiles of pigments extracted from *Synechocystis* strains lacking *chlB*

HPLC elution profiles of extracts from pellets of (A) Δ*chlB*, (B) Δ*bciB*/Δ*chlB* and (C) Δ*chlB*/Δ*bciB*::*bciA*^{Rs} strains after 16 h growth without illumination. Traces are normalized to major peak height for clarity.

[1] To whom correspondence should be addressed (email d.canniffe@sheffield.ac.uk).

© 2014 The Author(s)
This is an Open Access article distributed under the terms of the Creative Commons Attribution Licence (CC-BY) (http://creativecommons.org/licenses/by/3.0/) which permits unrestricted use, distribution and reproduction in any medium, provided the original work is properly cited.

Biochemical Journal www.biochemj.org

Table S1 List of strains and plasmids described in the present study

Strain/plasmid	Properties	Source
E. coli		
JM109	Cloning strain for pK18*mobsacB* and pIND4 constructs	Promega
S17-1	Conjugative strain for pK18*mobsacB* and pIND4 constructs	[1]
R. sphaeroides		
WT	2.4.1	S. Kaplan*
V3	Unmapped mutant in a DPOR subunit-encoding gene	[2]
V3::*bciB*^{Syn}	V3 harbouring pIND4[*bciB*]	The present study
V3/Δ*bciA* V3/Δ*bciA*::*bciB*^{Syn}	Unmarked deletion mutant of rsp_3070 in V3 V3/Δ*bciA* harbouring pIND4[*bciB*]	The present study
Δ*bchCXF*	Unmarked deletion mutant of *bchC, bchX* and *bchF* in WT	The present study
Δ*bchCXF*/Δ*bciA*	Unmarked deletion mutant of rsp_3070 in Δ*bchCXF*	The present study
Δ*bchCXF*::*bciB*^{Syn}	Δ*bchCXF* harbouring pIND4[*bciB*]	The present study
Δ*bchCXF*/Δ*bciA*::*bciB*^{Syn}	Δ*bchCXF*/Δ*bciA* harbouring pIND4[*bciB*]	The present study
Synechocystis		
WT	sp. PCC6803	R. Sobotka†
Δ*bciB*	*Em*^{R} replacement of central portion of *slr1923* in WT	[3]
Δ*bciB*::*bciA*^{Rs}	rsp_3070 and *Kan*^{R} replacement of *psbAII* in Δ*bciB*	[3]
Δ*chlB*	*Zeo*^{R} replacement of central portion of slr0772 in WT	The present study
Δ*bciB*/Δ*chlB*	*Zeo*^{R} replacement of central portion of slr0772 in Δ*bciB*	The present study
Δ*bciB*/Δ*chlB*::*bciA*^{Rs}	*Zeo*^{R} replacement of central portion of slr0772 in Δ*bciB*::*bciA*	The present study
Plasmid		
pK18*mobsacB*	Allelic exchange vector, *Km*^{R}	J. Armitage‡
pIND4	IPTG-inducible expression vector for *R. sphaeroides*, *Km*^{R}	[4]
pIND4[*bciB*]	slr1923 cloned into the BamHI/HindIII sites of pIND4	The present study

*Department of Microbiology and Molecular Genetics, The University of Texas Medical School, Houston, TX 77030, U.S.A.
†Institute of Microbiology, Department of Phototrophic Microorganisms, Opatovicky mlyn, 379 81 Trebon, Czech Republic
‡Department of Biochemistry, University of Oxford, South Parks Road, Oxford OX1 3QU, U.K.

© 2014 The Author(s)
This is an Open Access article distributed under the terms of the Creative Commons Attribution Licence (CC-BY) (http://creativecommons.org/licenses/by/3.0/) which permits unrestricted use, distribution and reproduction in any medium, provided the original work is properly cited.

Table S2 List of primers used in the present study

Restriction enzyme cleavage sites used for cloning are underlined in the primer sequence.

Primer	Sequence (5′→3′)	Cleavage site
rsp_3070UpF	CCGGAATTCGGACATCCTGACCGGTTTCCTGTCC	EcoRI
rsp_3070UpR	GCTCTAGAGGACATGGCGGAACTCCTCGGG	XbaI
rsp_3070DownF	GCTCTAGACGTTGACATTGGTGCCGGTCGG	XbaI
rsp_3070DownR	CCCAAGCTTCGAAGGCGATGCGCGCGAGGC	HindIII
rsp_3070CheckF	GACGACGAGAAGCTGGCCTACGG	
rsp_3070CheckR	GGCAGGTACCGGAGAGCGGTTAGG	
bchCXUpF	CCGGAATTCGCTCCTGCACCGGGTGCGCG	EcoRI
bchCXUpR	GCGCTCTAGAAGCGTTTTCCCCCGCGCTCTTC	XbaI
bchCXDownF	GCGCTCTAGATCTCGATACCCTCGCGCGGC	XbaI
bchCXDownR	CCCCAAGCTTGTCCTCGAACAGCTTGCCCGTG	HindIII
bchCXCheckF	GACGATCCACTGCCGCTCGG	
bchCXCheckR	CAGCGGCACGCCCGAGGCG	
bchFUpF	CCGGAATTCCCCCGCCCTGTCTCTCTGCAAGCC	EcoRI
bchFUpR	CGGGGGCGGAAGGTCAAGGCTCATCTTGAGGTTCGCCTTCCGAGGAGGGCCC	
bchFDownF	CCTACGTCATCAACGCCGGGCAGTTCC	
bchFDownR	CCCAAGCTTGCGCCGAGAGCCGCTCGGCCGCCCGG	HindIII
bchFCheckF	GACGGCCACCGGGCCCTC	
bchFCheckR	GAGGCGAGGCAGGCATCCTC	
1923INDF	CGCGGATCCACCGTTCCTGCCCCCCACC	BamHI
1923INDR	CCCAAGCTTTTATTGCTGGGGAAGTTTATACTGC	HindIII
chlBUpF	GCATCGCTTATTGTTCTCAACG	
chlBUpR	ACATTAATTGCGTTGCGCTCACTGCCGTGCATAATGGCGTGGACG	
chlBDownF	CAACTTAATCGCCTTGCAGCACATGCAGAATTGAATAAAGTGCCAGGG	
chlBDownR	CCTTCAAAGGCCATCACCC	
zeo^{R}F	TGACCAGCGCCGTTCCGGTG	
zeo^{R}R	CGGGTCGCGCAGGGCGAAC	

REFERENCES

1 Simon, R., Priefer, U. and Pühler, A. (1983) A broad host range mobilization system for *in vivo* genetic engineering: transposon mutagenesis in Gram negative bacteria. Nat. Biotechnol. **1**, 784–791 CrossRef

2 Nasrulhaq-Boyce, A., Griffiths, W. T. and Jones, O. T. G. (1987) The use of continuous assays to characterize the oxidative cyclase that synthesizes the chlorophyll isocycic ring. Biochem. J. **243**, 23–29 PubMed

3 Canniffe, D. P., Jackson, P. J., Hollingshead, S., Dickman, M. J. and Hunter, C. N. (2013) Identification of an 8-vinyl reductase involved in bacteriochlorophyll biosynthesis in *Rhodobacter sphaeroides* and evidence for the existence of a third distinct class of the enzyme. Biochem. J. **450**, 397–405 CrossRef PubMed

4 Ind, A. C., Porter, S. L., Brown, M. T., Byles, E. D., de Beyer, J. A., Godfrey, S. A. and Armitage, J. P. (2009) Inducible-expression plasmid for *Rhodobacter sphaeroides* and *Paracoccus denitrificans*. Appl. Environ. Microbiol. **75**, 6613–6615 CrossRef PubMed

Received 4 February 2014/2 June 2014; accepted 19 June 2014
Published as BJ Immediate Publication 19 June 2014, doi:10.1042/BJ20140163

© 2014 The Author(s)
This is an Open Access article distributed under the terms of the Creative Commons Attribution Licence (CC-BY) (http://creativecommons.org/licenses/by/3.0/) which permits unrestricted use, distribution and reproduction in any medium, provided the original work is properly cited.
